# Editorial: Musculoskeletal markers of healthy development in youth

**DOI:** 10.3389/fendo.2026.1916059

**Published:** 2026-08-06

**Authors:** Fátima Baptista, Kathleen F. Janz, Adam D. G. Baxter-Jones

**Affiliations:** 1 CIPER, Faculdade de Motricidade Humana, Universidade de Lisboa, Lisbon, Portugal; 2 Department of Epidemiology, University of Iowa, Iowa, IA, United States; 3 Department of Health and Human Physiology, University of Iowa, Iowa, IA, United States; 4 College of Kinesiology, University of Saskatchewan, Saskatoon, SK, Canada

**Keywords:** body composition, bone age assessment, bone health, bone turnover markers, functional hypothalamic amenorrhea, mechanical loading, musculoskeletal pain, skeletal maturation

The musculoskeletal system is fundamental to healthy growth and development in childhood and adolescence. Achieving optimal bone mass, skeletal maturation, muscle development, and body composition during these formative years has significant implications for lifelong health, influencing the risk of osteoporosis, fractures, and functional limitations later in life. Consequently, identifying reliable markers of musculoskeletal development has become a major focus of paediatric and lifespan research, reflecting growing recognition that many adult health outcomes have their origins in early-life development. This Research Topic, “*Musculoskeletal Markers of Healthy Development in Youth*”, brings together multidisciplinary contributions that advance our understanding of musculoskeletal health across early life. The articles address three interconnected themes: the assessment of skeletal development using innovative biomarkers and technologies; the biological and behavioural determinants of musculoskeletal health; and the clinical implications of early-life factors for future health and disease risk.

The review by Wang et al. highlights the growing role of bone turnover markers (BTMs) as dynamic, minimally invasive tools for assessing skeletal development in children and adolescents. Unlike imaging techniques, which provide a static assessment of bone mass, BTMs offer real-time insights into bone formation and resorption and may be particularly valuable for monitoring treatment responses and developmental trajectories. The authors also discuss their potential applications in paediatric bone and growth disorders. Importantly, the review emphasises key challenges that currently limit wider clinical implementation, including biological variability related to age, sex, maturation, and environmental influences, as well as methodological differences across assays and manufacturers.

In their original research article, Skaf et al. evaluated the performance of Deeplasia, an artificial intelligence-based system for automated bone age assessment, in children with rare endocrine, syndromic, and lysosomal storage disorders. Using a large multicentre dataset of more than 1,100 hand radiographs, the authors demonstrated that Deeplasia achieved high accuracy and consistency, often outperforming individual expert raters. These findings highlight the potential of artificial intelligence to enhance the objectivity, efficiency, and reproducibility of skeletal maturity assessment, particularly in clinically complex populations where conventional approaches may be challenged by atypical skeletal morphology and substantial inter-observer variability.

In their narrative review, Pepe et al. examine current approaches to assessing skeletal health from foetal life through early infancy, a critical period for skeletal development and the establishment of lifelong bone health. The authors review the strengths and limitations of available technologies, including DXA and quantitative ultrasound, and highlight the absence of standardised guidelines and validated reference values for this age group. Particular attention is given to Radiofrequency Echographic Multi-Spectrometry (REMS), an emerging, radiation-free ultrasound technology that offers the potential for safe, portable, and reproducible assessment of bone health in newborns and infants. The review underscores the urgent need for age-specific reference standards and longitudinal studies to support early detection, monitoring, and prevention strategies in paediatric bone health.

In a school setting, the measurement study by Brailey et al. supports observational and intervention-based research by quantifying vertical ground reaction forces and force-loading rates during everyday physical activities, including walking, running, and jumping. This work contributes to the limited body of research quantifying the characteristics of physical activity that influence musculoskeletal (MSK) health (1). It uniquely examines how force-loading varies by sex and biological maturity. As such, this paper may help inform researchers setting public health guidelines for bone-strengthening physical activity. It will also aid researchers in designing interventions to improve MSK health in youth.

Kardm et al. used a purposive sampling strategy to recruit youth with MSK pain, specifically those presenting to paediatric physiotherapy clinics. Using standardised and validated tests administered by licensed professionals, the researchers screened 683 children, of whom 550 met the inclusion criteria and completed a battery of behavioural and biomechanical assessments (2). The large sample size, *a priori* power calculation, and ability to measure a wide array of factors make this one of the more comprehensive observational studies of children’s MSK pain to date.

Using state-of-the-art measures to quantify the contributions of fat and lean mass to carotid and abdominal vascular function, the cross-sectional study by Evanoff et al. shows independent associations between tissue type and cardiovascular health. Their paper advances the field, most notably by using two vascular locations: the carotid artery and the abdominal aorta. In general, the carotid artery is most often used in paediatric research; yet the more internal abdominal aorta plays a large role in buffering arterial pressure and may provide a better predictor of early dysfunction. One of the authors’ novel findings suggests that lean mass is more protective of abdominal aorta function than of carotid artery function.

Together, the articles in this Research Topic highlight the importance of integrating advances in skeletal assessment with a deeper understanding of the biological and behavioural determinants of musculoskeletal health. Emerging biomarkers, innovative imaging technologies, and artificial intelligence are improving our ability to assess skeletal development, while studies of body composition, mechanical loading, physical activity, and vascular health provide valuable insights into the factors shaping musculoskeletal growth and lifelong health outcomes. These links between assessment, development, and future health are particularly evident in the review by Wong et al., which shows that functional hypothalamic amenorrhoea in adolescent female athletes can impair peak bone mass acquisition during a critical period of skeletal development, thereby increasing future fracture risk.

Collectively, these studies reinforce the importance of early identification, monitoring, and targeted interventions to optimise musculoskeletal development. Future research should continue to refine assessment tools and explore the complex interactions among skeletal, muscular, hormonal, and behavioural factors that shape musculoskeletal health across the life course. In addition to skeletal outcomes, greater attention should be paid to identifying early markers of muscle mass, strength, and power development, given their potential relevance to lifelong musculoskeletal function and health.
